# Performance Degradation Mechanism of Hemp Fiber-Reinforced Polypropylene Composites Under Accelerated Aging

**DOI:** 10.3390/polym17243309

**Published:** 2025-12-14

**Authors:** Wei Guo, Xiaorui Liu, Feng Zhao, Huayao Huang, Bo Li

**Affiliations:** 1 State Key Laboratory of Light Superalloys, Wuhan University of Technology, Wuhan 430070, China; whutgw@whut.edu.cn (W.G.); 13922312315@163.com (X.L.);; 2 Hubei Key Laboratory of Advanced Technology for Automotive Components, Wuhan University of Technology, Wuhan 430070, China; 3 Guangzhou Automobile Group Co., Ltd. (GAC), Honda Automobile Research & Development Co., Ltd., Guangzhou 511300, China; 4 Guangzhou Automobile Group Co., Ltd. (GAC), Automotive Research and Development Center, Guangzhou 511434, China

**Keywords:** hemp fiber, environmental aging, microstructure, performance degradationinjection molding

## Abstract

In the context of increasing resource scarcity and environmental concerns, the development of green composite materials is essential for promoting sustainability in the automotive industry. However, poor interfacial compatibility between plant fibers and polypropylene (PP), as well as the performance deterioration under complex environmental aging conditions, severely limits their engineering applications. In this study, a synergistic interfacial modification strategy combining alkali treatment of hemp fibers (HFs) with polypropylene grafted maleic anhydride (PP-g-MAH) was employed to enhance fiber–matrix interaction. Hemp fiber-reinforced polypropylene composites (HFRPs) with varying fiber contents (7.5–30 wt%) were fabricated via injection molding. Accelerated aging tests were conducted on the compatibilized HFRPs for up to 2400 h under ultraviolet–thermal–moisture coupled conditions, in accordance with the SAE J2527 standard. The evolution of surface color, mechanical properties, chemical structure, and microstructure was systematically characterized. After aging, surface whitening of the composites was observed. Tensile strength and impact strength decreased by 9.57–22.12% and 38.68–46.03%, respectively, while flexural strength remained relatively stable due to the supporting effect of the fiber skeleton. The aging of compatibilized HFRPs follows an outside-in progressive degradation mechanism, characterized by a stepwise cascade of surface oxidation, crack propagation, moisture ingress, interfacial degradation, and mechanical performance deterioration. These findings offer valuable insights into the long-term durability of natural fiber-reinforced thermoplastic composites and provide theoretical and practical guidance for their structural design and application in demanding service environments.

## 1. Introduction

Against the backdrop of increasingly severe resource depletion and environmental degradation, the development of plant fiber-reinforced composites (PFRCs) has become a research focus and important development direction in the field of materials science [1]. PFRCs are composite systems that use natural plant fibers (such as hemp [2], cotton [3], coconut shell [4], and bamboo fibers [5]) as the reinforcement phase and thermoplastic or thermosetting polymers as the matrix. These materials possess significant advantages, including low density, excellent renewability, low processing energy consumption, and strong biodegradability, exhibiting a promising trend of gradually replacing traditional materials in various fields [6,7].

In the automotive industry, polypropylene (PP) has become one of the most widely used matrix materials due to its excellent mechanical properties, good heat resistance, low cost, and superior processability and moldability [8]. Traditional PP composites usually adopt mineral fillers such as talc powder and calcium carbonate to enhance their rigidity and heat resistance [9,10]. However, such mineral fillers have an inherent defect of non-renewability and are prone to generating severe dust pollution during processing. Therefore, the replacement of mineral fillers with plant fibers has become an ideal solution. Among them, hemp fibers (HFs), with their outstanding mechanical properties, have shown significant application potential in the preparation of automotive interior parts, door panels, instrument panel substrates, and other components [11,12,13].

Due to the significant differences in polarity and structure between plant fibers and polymers, there exists a distinct interfacial compatibility issue during their compounding process [14,15]. Common interfacial modification strategies include physical roughening of the fiber surface [16], chemical grafting with coupling agents [17], plasma surface treatment [18], and the introduction of polar functional groups [19]. However, single modification methods are often insufficient to achieve strong and durable interfacial adhesion. In this study, a synergistic approach combining alkali treatment with polypropylene grafted maleic anhydride (PP-g-MAH) was adopted. The alkali treatment removes surface waxes, pectins, and portions of hemicellulose from the HFs, increasing surface roughness and specific surface area, while also exposing more reactive polar groups. These changes promote both mechanical interlocking and chemical bonding between the fiber and the polymer matrix [16,20]. Subsequently, PP-g-MAH was introduced as a compatibilizer. The maleic anhydride groups react with the hydroxyl groups on the fiber surface through esterification, thereby enhancing the interfacial adhesion and forming a stable and continuous interphase structure. This dual-modification strategy significantly improves the mechanical integrity and durability of the resulting composites [14,21].

Despite the favorable overall performance and ecological advantages of PFRCs, their environmental aging behavior remains a critical bottleneck limiting broader engineering applications. The organic nature of plant fibers and the physicochemical characteristics of the PP matrix make PFRCs particularly susceptible to performance degradation under complex service conditions involving ultraviolet (UV) radiation, heat, and moisture [7]. Recent studies have begun to explore the aging behavior of plant fiber-reinforced thermoplastic composites. It has been reported that UV exposure can trigger free-radical chain scission reactions in the PP matrix, leading to the formation of oxidative products, reduced molecular weight, and deteriorated mechanical properties [21]. In parallel, moisture uptake by plant fibers causes swelling, which can induce microcracks and interfacial debonding at the fiber–matrix interface. In severe cases, this can result in the disintegration of the reinforcement architecture [2]. Compared with pure water, saline water has been shown to exert a more pronounced aging effect, as composites immersed in saline solutions tend to absorb more moisture [22]. Among the various aging modes, hydrothermal aging is particularly significant for plant fibers. Alternating thermal and humid conditions accelerate interfacial degradation and structural loosening, further exacerbating performance loss. Elevated temperatures enhance the mobility of water molecules, promoting their diffusion into both the polymer matrix and the fiber interior [23]. These aging processes not only lead to visible changes in surface color and morphology but also result in substantial macroscopic deterioration of mechanical performance. However, most existing studies have focused on individual aging factors, which do not accurately reflect the multi-factorial environmental conditions encountered in real-world applications. In practice, PFRCs are typically subjected to coupled aging effects involving UV radiation, heat, and humidity. Yet, the underlying mechanisms governing the degradation of PFRCs under such synergistic aging conditions remain largely unexplored.

In this study, a series of hemp fiber-reinforced polypropylene composites (HFRPs) was fabricated via injection molding, using HFs as the reinforcement phase and PP as the matrix. The aging behavior and performance evolution of the composites were systematically investigated under the combined effects of ultraviolet (UV) radiation, moisture, and thermal exposure. Specifically, composites with varying HF contents (7.5 wt%, 15 wt%, 22.5 wt%, and 30 wt%) were subjected to accelerated climate aging tests for up to 2400 h under simulated environmental conditions. Comprehensive characterization was conducted to evaluate colorimetric changes (ΔE), mechanical properties (tensile, flexural, and impact strength), and morphological evolution on both the surface and fracture cross-sections. Moreover, the study elucidates the correlations among interfacial degradation, microstructural transformation, and macroscopic property deterioration during the aging process. This work provides a systematic experimental foundation for assessing the durability of natural fiber-reinforced thermoplastic composites under real-world service conditions. It also offers theoretical insights and practical guidance for material modification and structural design optimization, thereby advancing the application of PFRCs in long-term service environments such as the automotive industry.

## 2. Materials and Methods

### 2.1. Materials

The HFRPs used in this study were formulated from HFs, PP, and several processing additives. The PP resin (PP-SE1450H) was provided by CNOOC Shell Petrochemical Co., Ltd. (Huizhou, China). HFs were supplied by Remay Advanced Materials Co., Ltd. (Dongguan, China) and subsequently milled into short fibers with an average diameter of approximately 15 μm and a length of ~200 μm. For surface modification, sodium hydroxide (NaOH) and acetic acid (CH_3_COOH) were purchased from Hubei Zhongshui Chemical Co., Ltd. (Xiaogan, China). PP-g-MAH, serving as a compatibilizer, was obtained from Beijing Petrochemical Group Co., Ltd. (Beijing, China). Antioxidant B225 and calcium stearate (Cast), used as processing stabilizers, were purchased from Nanjing Jinling Chemical Plant Co., Ltd. (Nanjing, China).

### 2.2. Sample Preparation

**Figure 1:**
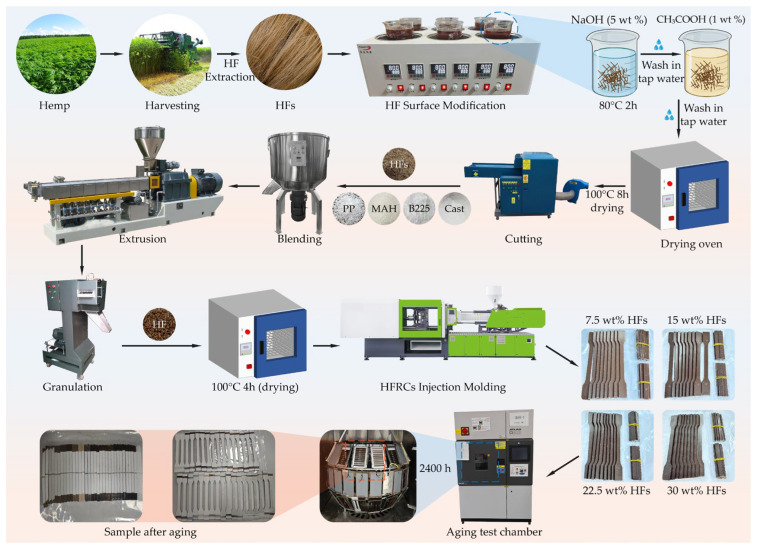
Schematic of HFs surface modification, HFRPs specimen fabrication, and accelerated aging procedure.

The fabrication process of HFRPs is illustrated in Figure 1. Mature hemp stalks were mechanically harvested and subjected to high-temperature boiling to extract HFs. The obtained fibers were immersed in a 5 wt% NaOH solution at 80 °C for 2 h to remove surface impurities, including hemicellulose, lignin, pectin, and waxes. After the alkaline treatment, the HFs were rinsed thoroughly with running water, followed by immersion in a 1 wt% CH_3_COOH solution to neutralize any residual NaOH. The fibers were then rinsed again with deionized water until reaching a neutral pH. The neutralized HFs were dried in a convection oven at 100 °C for 8 h and subsequently ground into short fibers with an average length of approximately 200 μm. According to the formulation detailed in Table 1, HFs, PP, PP-g-MAH, B225, and Cast were premixed using a high-speed mixer. The blend was then compounded using a twin-screw extruder (SHJ-20, Nanjing Jute Machinery Co., Ltd., Nanjing, China) operating at a screw speed of 250 rpm and a feeding rate of 550 rpm. The temperature profile from barrel zones I–IV and the die head was maintained at 185, 190, 190, 190, and 190 °C, respectively. The extruded strands were pelletized and dried at 100 °C for 4 h. Test specimens were prepared by injection molding (HDX50, Ningbo Haida Plastic Machinery Co., Ltd., Ningbo, China) under a temperature profile of 180, 190, 190, and 180 °C for barrel zones I–IV. The molded HFRP specimens were then subjected to accelerated environmental aging for up to 2400 h using a climate aging chamber (QUV/Spraze, Q-Panel Lab Products, Cleveland, OH, USA), under combined ultraviolet (UV), heat, and moisture exposure conditions. After the aging test, the samples were immediately taken out and placed in a vacuum drying oven for 24 h at 60 °C and −0.09 MPa to completely remove the moisture adsorbed on the sample surface and the free water permeated inside. After drying, the samples were placed in a standard laboratory environment (23 °C, relative humidity 50%) for equilibration for 4 h before conducting mechanical property tests, so as to avoid the interference of moisture on the test results.

### 2.3. Aging Tests

The accelerated aging tests for HFRPs were conducted in accordance with the SAE J2527 standard, which simulates long-term service conditions involving UV radiation, elevated temperatures, humidity, and water spray. A xenon arc lamp with extended UV output was employed as the light source, featuring a peak emission wavelength centered at 340 nm and an irradiance intensity of 0.55 W/(m^2^·nm). This setup effectively replicates the high-energy UV components of natural sunlight that contribute to the photodegradation of polymeric materials. Four aging durations were selected: 600, 1200, 1800, and 2400 h, in order to systematically evaluate the evolution of material properties and microstructure across different aging stages. The detailed aging cycle and environmental parameters are summarized in Table 2, which combines UV exposure with alternating thermal and humidity conditions to reflect realistic outdoor degradation scenarios.

The SAE J2527 standard simulates the outdoor automotive exposure environment through ultraviolet irradiation-condensation cycles. Its Acceleration Factor (AF) is estimated based on the principle of linear correlation between material aging rate and environmental stress intensity. According to classic research in the field of automotive material aging, the acceleration factor of the SAE J2527 standard is approximately 8–12 (i.e., 1 h of accelerated aging is equivalent to 8–12 h of outdoor natural exposure), a value that has been verified by comparing long-term outdoor exposure test data with accelerated aging data.

### 2.4. Characterization

#### 2.4.1. Mechanical Properties

Mechanical performance tests were conducted under ambient conditions. Tensile properties were evaluated according to GB/T 1040 using a universal testing machine with a loading rate of 20 mm/min, and the ultimate tensile strength was recorded. Flexural properties were assessed in accordance with GB/T 9341 under a loading rate of 2 mm/min to determine the flexural strength under static loading. Impact resistance was tested using a cantilever beam impact tester following GB/T 1843. The impact energy was set at 2.75 J, and standard V-notched specimens were used to evaluate the material’s resistance to brittle fracture.

#### 2.4.2. Fourier Transform Infrared Spectroscopy (FTIR)

The chemical structure of the composites was analyzed using Fourier transform infrared spectroscopy in the range of 400–4000 cm^−1^ with a resolution of 2 cm^−1^. This allowed for the identification of functional group variations before and after aging.

The carbonyl index (CI) is used to characterize the relative content of carbonyl groups in the material, which can effectively and quantitatively analyze the aging degree and stability of the material. Additionally, the correlation between microstructural changes and macroscopic performance degradation is established through the CI. The calculation method of CI is shown in Equation (1):

(1) $$CI=\frac{A_{C=0}}{A_{\mathrm{ref}}}\times k$$

In the equation: $A_{C=O}$ denotes the integral area of the complete carbonyl peak interval; $A_{ref}$ denotes the integral area of the complete internal standard peak interval; k is the correction coefficient, which is defaulted to 1 in this study.

#### 2.4.3. X-Ray Diffractometer (XRD)

Crystallographic properties of the composites were characterized using XRD. The measurements were performed under an accelerating voltage of 40 kV and a tube current of 40 mA. The scanning was carried out over a 2θ range of 5° to 80° with a step size of 0.05° and a scanning speed of 1°/min to analyze phase composition and crystallinity evolution.

The crystallinity (X_n_) of the composite material is calculated to quantitatively characterize the proportion of crystalline phases within the material, thereby better analyzing the aging mechanism and evaluating the material’s stability. The calculation method of X_n_ is shown in Equation (2):

(2) $$\chi_{n}=\frac{A_{C}}{A_{C}+A_{a}}\times 100\%$$

In the equation: $A_{C}$ and $A_{a}$ represent the integral areas of the crystalline peak and the amorphous peak, respectively. Given that the crystalline peak and amorphous peak of HPFRs partially overlap with relatively complex peak shapes, the peak splitting fitting method is adopted in this study to calculate the integral areas of the crystalline peak and the amorphous peak separately.

#### 2.4.4. Surface Color Change

Surface color variations in the samples were measured using a colorimeter based on the CIELAB color space system. The L (lightness), a (red–green), and b (yellow–blue) parameters were recorded, and the total color difference (ΔE) before and after aging was calculated to assess visual appearance degradation.

#### 2.4.5. Hardness

The surface hardness of the composites before and after aging was measured using a Shore D hardness tester. Care was taken to ensure flat and defect-free surfaces during testing to enhance reproducibility and accuracy.

#### 2.4.6. Scanning Electron Microscope (SEM)

To observe microstructural features and fracture morphology after aging, samples were pre-conditioned in liquid nitrogen for 10 min and then cryo-fractured. The fractured surfaces were sputter-coated with a thin layer of platinum to enhance conductivity and imaged using SEM at an accelerating voltage of 15 kV. The midsection of standard specimens was selected as the observation region to ensure consistency and representativeness.

## 3. Results and Discussion

### 3.1. Surface Modification of HFs

**Figure 2:**
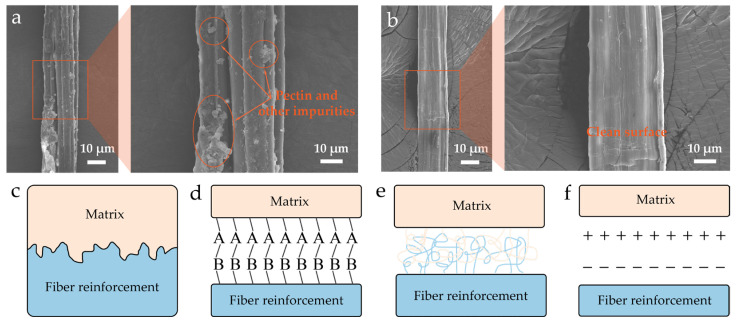
(**a**) Surface morphology of untreated HFs. (**b**) Surface morphology of HFs after NaOH treatment. (**c**–**f**) Schematic illustration of interfacial bonding mechanisms in the composite: (**c**) mechanical interlocking, (**d**) chemical bonding, (**e**) molecular diffusion, and (**f**) electrostatic interaction.

The surface morphology of HFs underwent substantial changes after alkaline treatment, as shown in Figure 2a,b. Both the structural and chemical characteristics of the fiber surface were significantly modified, thereby influencing its reinforcing effect in the composite. SEM images clearly revealed that untreated HFs’ surfaces were covered with a large amount of pectin, wax, and other non-cellulosic impurities. This resulted in a smooth, hydrophobic surface, which hindered effective interfacial bonding with the PP matrix. The core mechanism of NaOH alkaline treatment for plant fibers lies in alkaline hydrolysis. It disrupts the chemical structure of the lignin hemicellulose complex on the fiber surface, enabling the selective removal of hemicellulose, lignin, and surface attachments. This process not only effectively exposes the cellulose main chains and crystalline structures but also induces the formation of distinct groove-like textures on the fiber surface, thereby significantly enhancing the fiber’s surface roughness and specific surface area [24,25,26]. These resulting topographical features enhance the mechanical interlocking between HFs and the PP matrix, facilitating the embedding of polymer chains into the fiber grooves during melt processing and thus promoting a more stable physical interface [14]. In addition, the binding substances on the fiber surface are removed, and the originally aggregated fiber bundles disaggregate. This leads to a decrease in the average fiber diameter, a more uniform size distribution, and a slight increase in the aspect ratio [27,28]. Moreover, the exposure of cellulose introduced more reactive sites—such as –OH groups—on the fiber surface, increasing opportunities for molecular interactions. The elevated surface energy further improved interfacial wettability and compatibility with the PP matrix. These structural changes not only enhanced fiber–matrix wetting behavior but also facilitated esterification reactions between the hydroxyl groups on HFs and the anhydride groups in PP-g-MAH. This interfacial chemical bonding significantly strengthened the adhesion between the fiber and matrix, ultimately improving the interfacial compatibility and mechanical integrity of the composite.

As illustrated in Figure 2c, interfacial bonding in fiber-reinforced composites typically involves multiple mechanisms, including mechanical interlocking, chemical bonding, molecular diffusion, and electrostatic attraction [29]. Alkaline treatment enhances the interfacial adhesion on both the physical (morphological) and chemical (reactive site) levels, thereby contributing to improved mechanical stability and structural integrity of the composites under long-term aging conditions. These interfacial reinforcement mechanisms play a crucial role in resisting microcrack propagation and maintaining interfacial stability during environmental aging. They are considered essential for improving the long-term durability of the material [30] (Further details are shown in Figure S1).

### 3.2. Color Changes on the Surface of the Aged Sample

**Figure 3:**
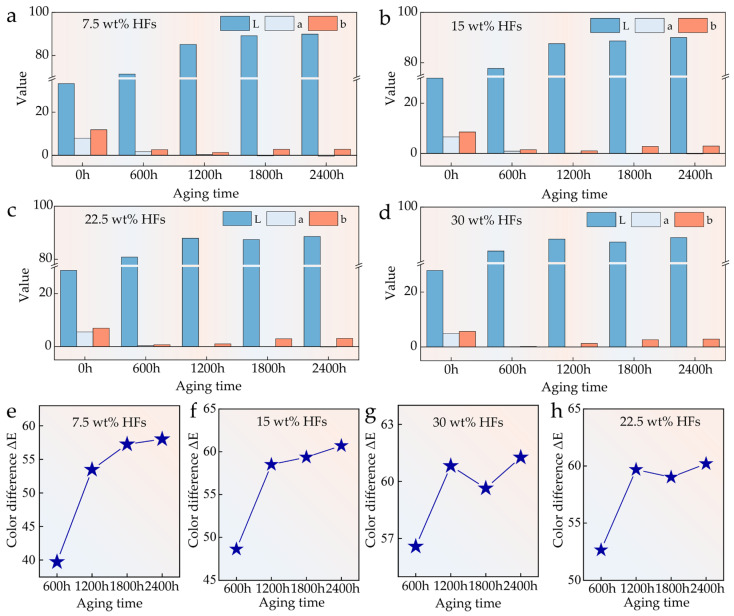
Surface color changes of HFRPs with varying HF content after aging: (**a**) 7.5 wt%, (**b**) 15 wt%, (**c**) 22.5 wt%, (**d**) 30 wt%. Comparison of surface color difference (ΔE) of aged HFRPs at different fiber contents: (**e**) 7.5 wt%, (**f**) 15 wt%, (**g**) 22.5 wt%, (**h**) 30 wt%.

After long-term aging, HFRPs exhibited a pronounced surface whitening phenomenon that was clearly visible to the naked eye (Figure 1). To quantitatively assess this change, surface color measurements were conducted using a colorimeter, and the results are presented in Figure 3. In the CIELAB color space, the L, a, and b values represent lightness, red–green axis, and yellow–blue axis, respectively, while ΔE quantifies the total color difference before and after aging. Before aging, the L, a, and b values of the composite surface showed a slight decrease with increasing HF content, primarily due to the inherent masking effect of the fiber’s natural color. Upon aging, a substantial increase in L value and a significant reduction in a and b values were observed. After 600 h of aging, the L value more than doubled, while both a and b values approached zero. Beyond 1200 h of exposure, the surface color parameters tended to stabilize, and the a value could even shift to negative, indicating a transition toward a greenish hue. These color changes are attributed to UV-induced photooxidation, which leads to surface microporosity and chemical bleaching. The degradation of chromophoric groups results in reduced color saturation and increased whiteness of the surface [31]. With higher HF content, the proportion of natural components in the composite increases, making the material more sensitive to UV radiation and moisture. Consequently, under identical aging conditions, composites with higher fiber content exhibit more pronounced changes in ΔL, Δa, and Δb—corresponding to greater degrees of surface whitening and fading. Statistical analysis of surface color differences (Figure 2e,f) further confirms this trend. The most significant changes in ΔE occurred during the initial aging phase (0–600 h), after which the surface color gradually stabilized. These results demonstrate the dynamic evolution and eventual equilibrium of surface color under prolonged environmental exposure.

### 3.3. Hardness Changes on the Surface of the Aged Sample

**Figure 4:**
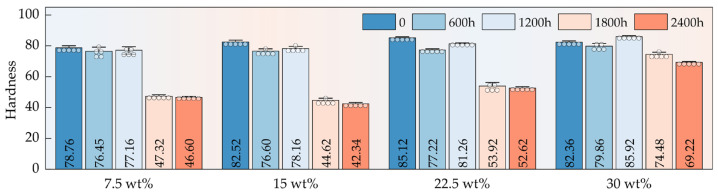
Surface hardness evolution of HFRPs as a function of HF content and aging duration.

The surface hardness of HFRPs is significantly influenced by both aging duration and HF content. As shown in Figure 4, before aging, the surface hardness of the composites gradually increased with HF content, rising from 7.5 wt% to 30 wt%. However, the overall increase was relatively modest, which is primarily attributed to the inherent hardness contrast between HFs and the PP matrix [32,33]. Due to its inherent stiffness, HFs contribute to resistance to deformation and delay the progression of hardness loss. Notably, with increasing aging duration, the composite’s hardness exhibited a pronounced downward trend. In particular, after 1800 h of exposure, the surface hardness showed a sharp decline, indicating catastrophic structural degradation. This sudden drop is primarily caused by the cumulative effects of UV radiation and hydrothermal aging, which severely erode the PP matrix at the surface, break molecular chains, reduce polymerization degree, and rapidly diminish mechanical strength.

### 3.4. FTIR and XRD Analysis

**Figure 5:**
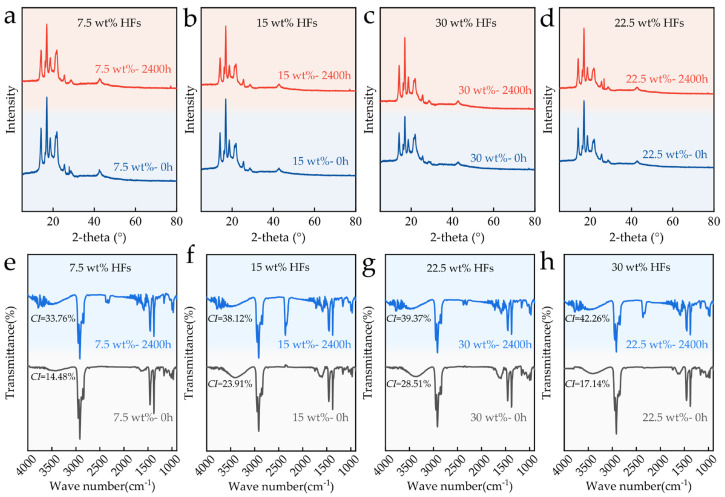
XRD patterns of HFRPs with varying HF contents before and after aging: (**a**) 7.5 wt%, (**b**) 15 wt%, (**c**) 22.5 wt%, (**d**) 30 wt%. FTIR spectra of HFRPs with varying HF contents before and after aging: (**e**) 7.5 wt%, (**f**) 15 wt%, (**g**) 22.5 wt%, (**h**) 30 wt%.

Figure 5a–d presents the XRD patterns of HFRP specimens with different HF contents before and after aging. In the unaged state (0 h), all samples exhibited characteristic crystalline peaks of PP located at approximately 14.1°, 16.9°, 18.6°, and 21.3°, consistent with α-phase PP crystallites [34]. As HF content increased, a slight enhancement in peak intensity was observed, and the crystallinity also increased accordingly, suggesting that the presence of fibers may promote localized crystallization of the PP matrix through a nucleating effect. After 2400 h of aging, the main diffraction peaks of the PP matrix showed significant intensity reduction and broadening, especially at 14.1° and 21.3°, and the total crystallinity of the composite material decreased, indicating progressive disruption of the crystalline structure and increased amorphization. Samples with higher HF content (e.g., 30 wt%) retained more pronounced crystalline peaks, and the degree of crystallinity reduction is smaller, suggesting that plant fibers can partially protect the crystalline regions of PP during environmental degradation. Nonetheless, aging induced a general transformation of the matrix from a highly ordered crystalline state to a more disordered, amorphous structure, reflecting the destructive impact of UV radiation and thermal–oxidative stress on PP crystallinity.

Figure 5e–h displays the FTIR spectra of the same series of HFRPs before and after aging. In the unaged condition, all samples exhibited typical PP absorption bands: C-H stretching vibrations near 2950 cm^−1^, 2916 cm^−1^, and 2845 cm^−1^; CH_3_ bending at 1454 cm^−1^ and 1376 cm^−1^; and C-C and C-CH_3_ vibrations at 1166 cm^−1^ and 998 cm^−1^ [35]. As the HF content increased, the absorption band near 3400 cm^−1^—corresponding to O–H stretching—became more intense, indicating increased contributions from hemicellulose and cellulose components within the fibers [36]. Following 2400 h of aging, all samples exhibited noticeable enhancement or emergence of absorption bands at 1720 cm^−1^, attributed to C=O stretching vibrations from carbonyl groups. Meanwhile, the CI of the material also increases after aging, a hallmark of PP oxidative degradation. Additionally, the diffraction peak observed near 2400 cm^−1^ in the aged samples is likely due to the loosened surface structure and enhanced adsorption capacity, which may have led to the adsorption of trace amounts of CO_2_ or H_2_O from the ambient environment [31]. These peaks may also be related to increased surface porosity and adsorption of trace environmental CO_2_ or H_2_O. The overall weakening of PP-specific peaks (e.g., 2950 cm^−1^ and 1454 cm^−1^) further suggests partial scission of the polymer main chains and deterioration of the chemical structure.

### 3.5. Mechanical Property Changes of the Aged Samples

#### 3.5.1. Tensile Properties

**Figure 6:**
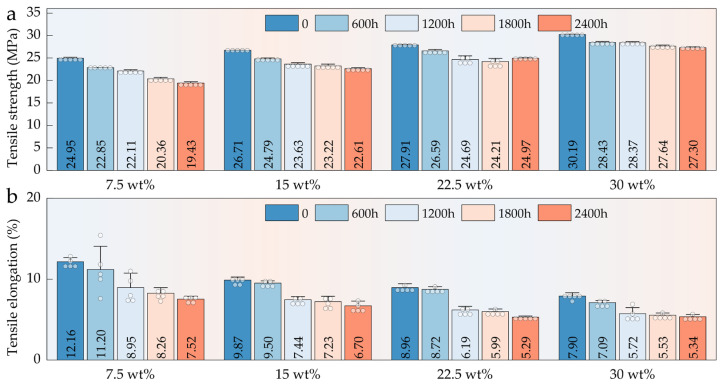
Tensile properties of HFRPs with varying HF content and aging durations: (**a**) tensile strength; (**b**) elongation at break.

The tensile properties of HFRPs progressively deteriorated with increasing aging time, as evidenced by continuous reductions in both tensile strength and elongation at break (Figure 6a,b). After 2400 h of aging, the tensile strength of samples with 7.5 wt%, 15 wt%, 22.5 wt%, and 30 wt% HF content decreased by 22.12%, 15.35%, 10.53%, and 9.57%, respectively. The addition of HFs effectively delayed the degradation of mechanical performance, particularly at higher fiber loadings. During the early stage of aging (0–1200 h), tensile strength declined sharply, suggesting that under the synergistic effects of UV radiation, heat, and moisture, severe degradation of the PP matrix occurred, leading to a rapid loss of mechanical integrity. After 1200 h, the rate of tensile strength reduction slowed. This transition is attributed to the formation of a surface oxidation layer, which, having undergone substantial degradation, acts as a physical barrier that mitigates further penetration of oxidative species, thereby slowing the degradation of the subsurface material. HFs, particularly at higher contents (22.5 wt% and 30 wt%), contributed to load-bearing even after surface matrix degradation, thus helping to delay structural failure. Compared to tensile strength, the degradation of elongation at break was more pronounced and occurred earlier. This is primarily because ductility is highly dependent on the integrity and flexibility of the PP matrix. During aging, chain scission events led to a significant loss of the matrix’s plastic deformation capacity. In combination with interfacial debonding and microcrack propagation, the fracture mode of the composite transitioned from ductile to brittle, resulting in a dramatic reduction in elongation at break, especially in the early stages of exposure.

#### 3.5.2. Flexural Properties

**Figure 7:**
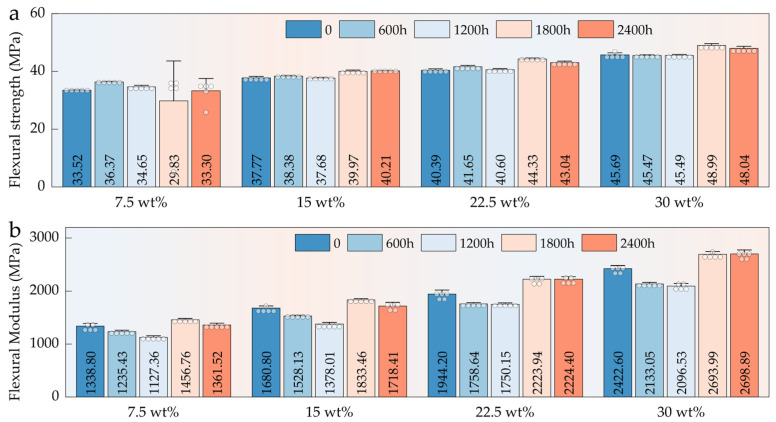
Flexural properties of HFRPs with varying HF content and aging durations: (**a**) flexural strength; (**b**) flexural modulus.

Compared with tensile performance, HFRPs exhibited superior retention of flexural properties during long-term aging (Figure 7a). Notably, in composites with higher HF contents (≥15 wt%), no significant decline in flexural strength was observed even after 2400 h of aging; instead, a slight increase was detected. This enhancement is closely associated with the load-bearing contribution of the fiber network. Dispersed HFs within the PP matrix act as a reinforcing skeleton, effectively resisting deformation under external loads. Under bending stress, the orientation and inherent stiffness of the fibers improve structural support, particularly in compression zones, thereby enhancing the overall flexural resistance. As shown in Figure 7b, the flexural modulus of HFRPs exhibited a trend of first decreasing and then increasing over the 0–2400 h aging period. Overall, the flexural modulus after 2400 h of aging was slightly higher than that of the unaged samples. This effect was more pronounced at fiber contents above 15 wt%, suggesting that sufficient HFs incorporation contributes to the stiffness and integrity of the composite structure, even under prolonged environmental degradation.

#### 3.5.3. Impact Properties

**Figure 8:**
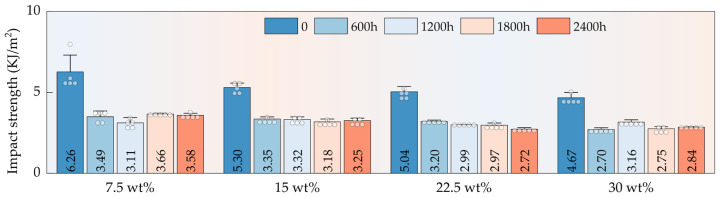
Impact strength of HFRPs as a function of HF content and aging time.

Under long-term environmental aging, HFRPs exhibited the most pronounced degradation in impact strength—greater than the reductions observed in tensile and flexural properties (Figure 8). After 2400 h of aging, the impact strength of composites with 7.5 wt%, 15 wt%, 22.5 wt%, and 30 wt% HF content decreased by 42.81%, 38.68%, 46.03%, and 39.19%, respectively. Impact strength reflects a material’s ability to resist sudden fracture under high-strain-rate conditions and is strongly dependent on matrix toughness, interfacial integrity, and energy dissipation mechanisms. During the early aging stages, exposure to UV radiation and combined thermal–humid environments induced oxidative chain scission in the PP matrix, drastically reducing molecular weight and compromising chain flexibility and extensibility. This led to a significant loss in matrix toughness, rendering the material more susceptible to brittle fracture under impact loading. After 600 h of aging, a steep drop in impact strength was observed, indicating that the critical degradation threshold had been reached. At this point, microstructural damage in both the matrix and interface facilitated crack initiation, and the failure mode shifted from ductile to brittle, resulting in catastrophic loss of impact resistance.

### 3.6. Microstructure Changes of the Aged Samples

#### 3.6.1. Surface Morphology

**Figure 9:**
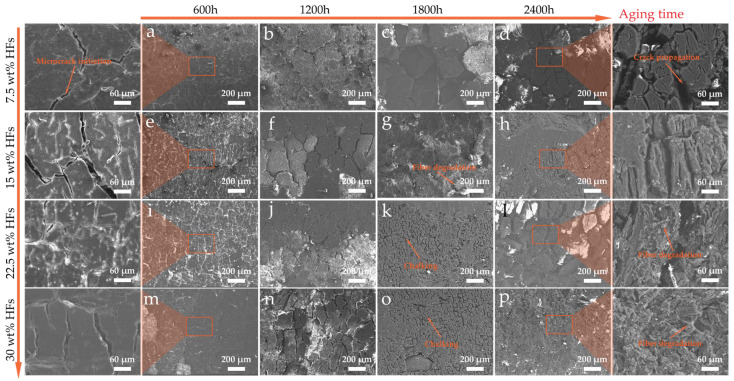
Surface microstructure evolution of HFRPs under long-term environmental aging: (**a**) 7.5 wt% HFs—600 h, (**b**) 7.5 wt% HFs—1200 h, (**c**) 7.5 wt% HFs—1800 h, (**d**) 7.5 wt% HFs—2400 h, (**e**) 15 wt% HFs—600 h, (**f**) 15 wt% HFs– 1200 h, (**g**) 15 wt% HFs—1800 h, (**h**) 15 wt% HFs—2400 h, (**i**) 22.5 wt% HFs—600 h, (**j**) 22.5 wt% HFs—1200 h, (**k**) 22.5 wt% HFs—1800 h, (**l**) 22.5 wt% HFs—2400 h, (**m**) 30 wt% HFs—600 h, (**n**) 30 wt% HFs—1200 h, (**o**) 30 wt% HFs—1800 h, (**p**) 30 wt% HFs—2400 h.

The evolution of surface micromorphology of HFRPs during long-term aging is illustrated in Figure 9. As aging time extends, significant deterioration occurs on the material surface. Specifically, the surface color gradually whitens from the initial state, microcracks initiate and continuously increase in quantity and depth, and further characteristics of surface chalking and peeling-off appear in the late aging stage. From the perspective of the aging process, scattered microcracks first emerge on the surface layer of the composite material after 600 h of aging. At this stage, the cracks are small and discrete, without forming a connected structure. With the continuous extension of aging time, the initially initiated microcracks gradually connect, converge, and propagate, eventually forming a continuous visible crack network on the surface layer, with a simultaneous increase in crack depth. After 2400 h of aging, the chalked surface layer begins to spall off, and the crack dimensions significantly enlarge, with the maximum crack width reaching approximately 50 μm. Some cracks have penetrated to the fiber–matrix interface region, leading to the failure of interface bonding and further exposing the HFs whose surfaces have been degraded or damaged. Notably, after long-term aging, all HFRP samples with different fiber contents form obvious crack structures on the surface layer, and no significant difference is observed in the overall crack size (width and depth). In addition, a high fiber content (≥15 wt%) increases the volume fraction of HFs in the composite material and enhances the distribution density of fibers in the surface region. Therefore, the exposure of HFs is more easily observed on the surface layer of samples with high fiber content, and the surface degradation and damage characteristics of the exposed fibers are more obvious. The surface degradation of HFRPs under the experimental conditions mainly originates from the chain scission, oxidation, and embrittlement of polypropylene (PP) under the thermal–oxygen–photochemical coupling effect, rather than the large-scale failure of the fiber itself. Fibers are passively exposed, and their damage is mostly induced by the deterioration of the surrounding matrix.

#### 3.6.2. Cross-Section Morphology

**Figure 10:**
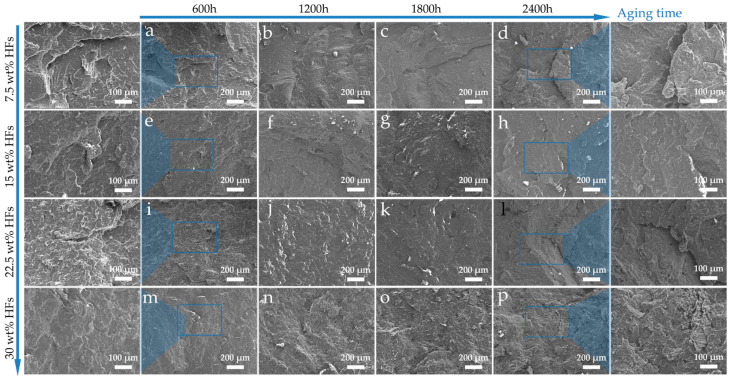
Cross-sectional microstructure of HFRPs after long-term environmental aging: (**a**) 7.5 wt% HFs—600 h, (**b**) 7.5 wt% HFs—1200 h, (**c**) 7.5 wt% HFs—1800 h, (**d**) 7.5 wt% HFs—2400 h, (**e**) 15 wt% HFs—600 h, (**f**) 15 wt% HFs– 1200 h, (**g**) 15 wt% HFs—1800 h, (**h**) 15 wt% HFs—2400 h, (**i**) 22.5 wt% HFs—600 h, (**j**) 22.5 wt% HFs—1200 h, (**k**) 22.5 wt% HFs—1800 h, (**l**) 22.5 wt% HFs—2400 h, (**m**) 30 wt% HFs—600 h, (**n**) 30 wt% HFs—1200 h, (**o**) 30 wt% HFs—1800 h, (**p**) 30 wt% HFs—2400 h.

As illustrated in Figure 10, the fracture surface micromorphology of composites after aging is presented, revealing the structural evolution characteristics of the material under long-term environmental stress. Detailed observation of the fracture surface reveals that HFs exhibit a typical random orientation pattern in the polypropylene (PP) matrix. With the increase in fiber mass fraction, the number of visible fibers on the fracture surface increases significantly. The fundamental reason for this phenomenon lies in the increase in the total fiber volume fraction, which makes the distribution of fibers denser across different spatial levels, thereby facilitating the exposure of the reinforcement phase from the fracture surface. Regarding the influence of the aging process on the fracture surface structure, as aging time extends, the microcracks generated in the surface layer of the composites due to the accumulation of environmental stress tend to gradually penetrate and propagate inward, but they do not penetrate deep into the bulk of the composites. Based on the fracture surface morphology characterization results, it is evident that regardless of the aging duration, the internal bulk structure of the composites remains relatively intact. No obvious through cracks, pores, fiber agglomeration degradation, or other phenomena are observed, and the integrity of the internal structure is maintained. The aforementioned microstructural evolution characteristics further clarify the intrinsic mechanism by which composites with high fiber content tend to retain their performance after long-term aging. The performance degradation of composites is mainly attributed to the degradation of the surface material and microcrack deterioration, while no substantial damage occurs to their internal bulk structure. For samples with high fiber content, the densely distributed HFs inside can form a performance compensation effect through their load transfer capacity, deformation resistance, and structural stability. The performance gain brought by these fibers can offset the adverse impact of surface degradation on the overall performance of the composites to a certain extent.

### 3.7. Aging Mechanism of HFRPs

**Figure 11:**
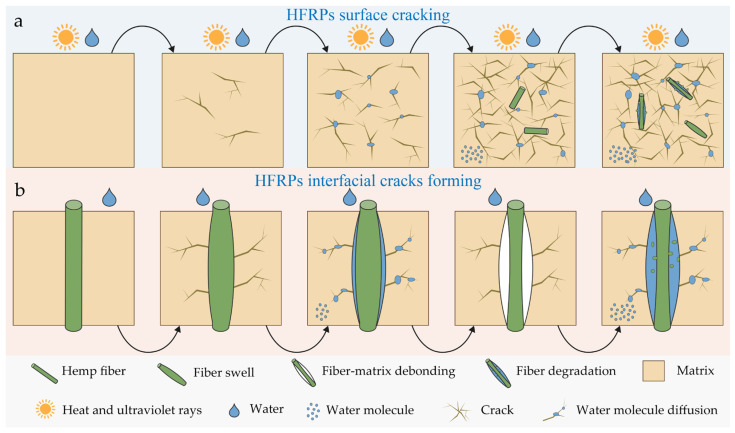
Schematic illustration of the long-term environmental aging mechanism of HFRPs: (**a**) surface crack initiation and propagation; (**b**) interfacial crack formation and propagation at the HFs–PP interface.

The aging behavior of HFRPs under multiple environmental stresses (e.g., ultraviolet radiation, heat, and moisture) exhibits an externally initiated composite degradation mechanism, as illustrated in Figure 11. In the early stage of aging, ultraviolet radiation serves as the dominant factor. Its energy is sufficient to induce polypropylene (PP) segment scission and free radical reactions, leading to the generation of oxidative groups on the material surface, which thereby alters the surface polarity and chemical structure of the material [37]. This process is accompanied by morphological changes such as surface whitening and microcrack initiation. Meanwhile, with the assistance of high temperature, the thermal motion of PP is enhanced, accelerating the rate of oxidation reactions and intensifying the surface degradation process. This is manifested as obvious surface shrinkage, embrittlement, and crack formation. As aging progresses, the number and depth of surface cracks increase significantly, eventually leading to the peeling of part of the surface material and exposing the HFs near the surface to the external environment. At this stage, water molecules further penetrate, and the hydro-thermal coupling-dominated degradation mechanism gradually becomes one of the dominant aging factors. Since HFs are natural porous materials with inherent hydrophilicity, their structure contains polar groups such as hydroxyls and hemicelluloses. After fiber exposure, water molecules can penetrate the material’s interior along the fiber channels. This penetration path exerts a dual effect: on one hand, it provides a rapid channel for moisture to enter the matrix interior; on the other hand, fiber swelling upon water absorption induces local expansion stress and changes in interfacial tension at the fiber–matrix interface, thereby initiating or exacerbating the formation of interfacial cracks. The surface layer of the composite evolves from a dense, tightly bonded reinforcement system with strong interfaces into a degraded structure characterized by multiple cracks, numerous voids, and weak interfaces. This results in a significant degradation of overall mechanical properties, especially sensitive indicators such as impact strength and tensile properties. However, limited by the aging duration, water molecules do not penetrate further into the material interior, allowing the integrity of the composite core to be maintained.

## 4. Conclusions

This study investigates the environmental degradation mechanisms and performance evolution of HFRPs through interfacial synergistic modification and multi-factor coupled aging experiments. The combined use of NaOH alkaline treatment and maleic anhydride-grafted polypropylene (PP-g-MAH) as a compatibilizer significantly enhanced the mechanical interlocking and chemical bonding between the fibers and the matrix, thereby establishing a foundation for improved long-term aging resistance. Under a combined UV–thermal–moisture aging environment, HFRPs exhibited surface whitening, increased and deepened microcracks, and exposed fibers. After aging, the tensile and impact strengths of the composites decreased by 9.57–22.12% and 38.68–46.03%, respectively, while the flexural strength remained relatively stable due to the load-bearing support provided by the fiber network. The incorporation of HFs partially mitigated the overall performance deterioration. Following 2400 h of aging, damage was primarily localized at the surface layer but showed a tendency to propagate inward, revealing a progressive degradation mechanism involving surface oxidation, crack propagation, moisture penetration, interfacial debonding, and consequent property decline. These findings provide valuable insights into the long-term service behavior of HFRPs and offer a scientific basis for their durability-oriented design and engineering applications in automotive and other structural fields.

## Figures and Tables

**Table 1:** Material formulations of HFRP specimens.

HFs (wt%)	PP (wt%)	PP-g-MAH (wt%)	B225 (wt%)	Cast (wt%)
7.5	91.1	1	0.2	0.2
15	82.6	2	0.2	0.2
22.5	74.1	3	0.2	0.2
30	65.6	4	0.2	0.2

**Table 2:** Parameters and cycle settings for the accelerated aging chamber.

Standard	SAE J2527 (Automotive)
BPT	70 °C
CAT	47 °C
Relative humidity in the dry phase	50%RH.
Work cycle	40 min dry/light
	20 min front-spray/light
	60 min dry/light
	60 min front- and back-spray/dark

## Data Availability

The original contributions presented in this study are included in the article/Supplementary Material. Further inquiries can be directed to the corresponding authors.

## Supplementary Materials

The following supporting information can be downloaded at: https://www.mdpi.com/article/10.3390/polym17243309/s1, Figure S1: (a) Surface morphology of untreated HF. (b) Surface morphology of HF following treatment with 2.5 wt% NaOH. (c) Surface morphology of HF following treatment with 5 wt% NaOH. (d) Surface morphology of HF following treatment with 7.5 wt% NaOH. (e) Surface morphology of HF following treatment with 10 wt% NaOH.
