# Supplementary material for: Performance Degradation Mechanism of Hemp Fiber-Reinforced Polypropylene Composites Under Accelerated Aging
Source: Polymers (Basel). 2025 Dec 14;17(24):3309. doi: 10.3390/polym17243309 (PMC12736921; doi:10.3390/polym17243309)
Supplement: Supplementary file 1 [file polymers-17-03309-s001.zip › polymers-4018151-supplementary.pdf]

Performance Degradation Mechanism of Hemp Fiber-Reinforced Polypropylene Composites under Accelerated Aging

Wei Guo[1,2], Xiaorui Liu[1,2,3], Feng Zhao[1,2,*] Huayao Huang[1,2], and Bo Li[4,*]

[1] State Key Laboratory of Light Superalloys, Wuhan University of Technology, Wuhan 430070, China;
[2] Hubei Key Laboratory of Advanced Technology for Automotive Components, Wuhan University of Technology, Wuhan, China;
[3] GAC Honda Automobile Research & Development Co., Ltd., Guangzhou, China;
[4] GAC Automotive Research and Development Center, Guangzhou, China;
* Correspondence: zhaof@whut.edu.cn (F.Z.); libo1@gacrnd.com (B.L.)

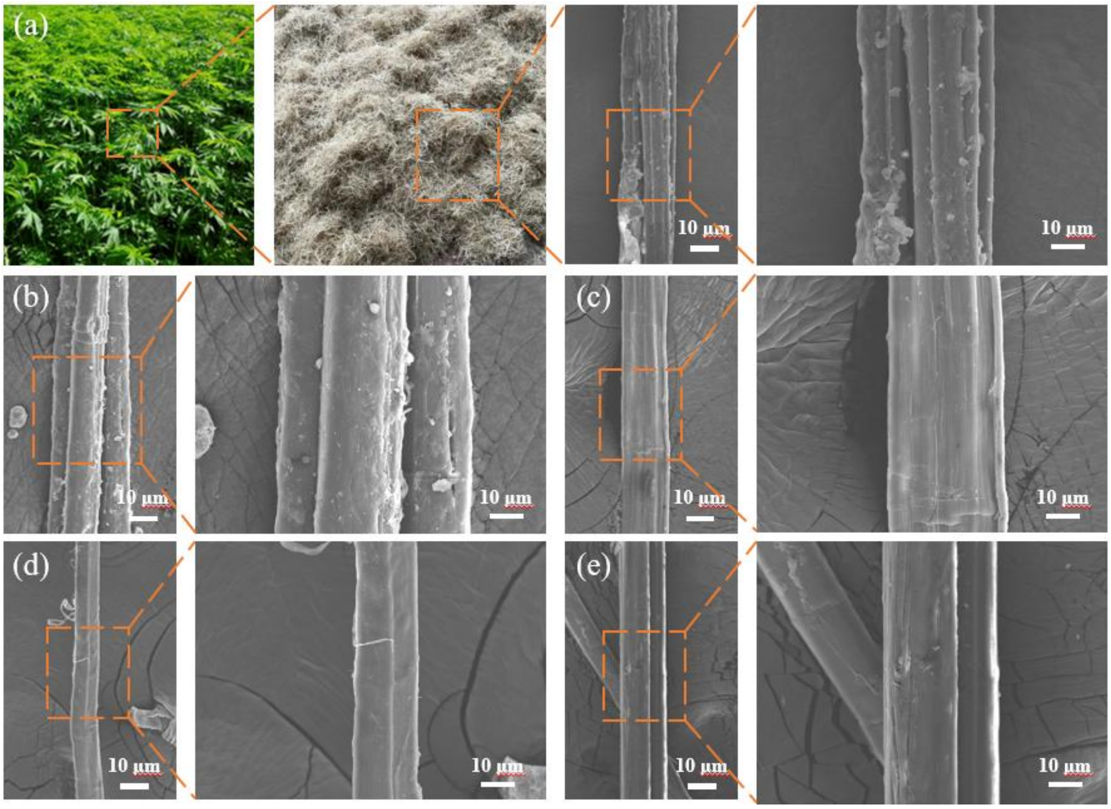

Figure S1. (a) Surface morphology of untreated HF. (b) Surface morphology of HF following treatment with 2.5 wt% NaOH. (c) Surface morphology of HF following treatment with 5 wt% NaOH. (d) Surface morphology of HF following treatment with 7.5 wt% NaOH. (e) Surface morphology of HF following treatment with 10 wt% NaOH.
